# Deep Sea Actinomycetes and Their Secondary Metabolites

**DOI:** 10.3389/fmicb.2017.00760

**Published:** 2017-05-01

**Authors:** Manita Kamjam, Periyasamy Sivalingam, Zinxin Deng, Kui Hong

**Affiliations:** Key Laboratory of Combinatorial Biosynthesis and Drug Discovery, Ministry of Education, Wuhan University School of Pharmaceutical SciencesWuhan, China

**Keywords:** deep sea, actinomycetes, bioactive natural products, biosynthesis, novel species

## Abstract

Deep sea is a unique and extreme environment. It is a hot spot for hunting marine actinomycetes resources and secondary metabolites. The novel deep sea actinomycete species reported from 2006 to 2016 including 21 species under 13 genera with the maximum number from *Microbacterium*, followed by *Dermacoccus, Streptomyces* and *Verrucosispora*, and one novel species for the other 9 genera. Eight genera of actinomycetes were reported to produce secondary metabolites, among which *Streptomyces* is the richest producer. Most of the compounds produced by the deep sea actinomycetes presented antimicrobial and anti-cancer cell activities. Gene clusters related to biosynthesis of desotamide, heronamide, and lobophorin have been identified from the deep sea derived *Streptomyces*.

## Introduction

The search and discovery of novel microbes that produce new secondary metabolites can be expected to remain significant in the race against new and emerging diseases and antibiotic resistant pathogens (Berdy, 2012; Manivasagan et al., 2013). Actinomycetes are widely distributed in various marine habitats, ranging from sea sand (Hong et al., 2008), mangrove sediments (Hong et al., 2009; Hong, 2013; Azman et al., 2015), sea water (Zhang L. et al., 2012), coastal sediments (Yu et al., 2015), and deep sea sediments (Zhang et al., 2015; Chen et al., 2016). The increasing number of literature on marine actinomycetes strongly supported the view that marine environments including deep sea are significant sources for search and discovery of both diverse actinomycetes resources and secondary metabolites (Skropeta and Wei, 2014; Xu et al., 2014).

Deep sea habitats show extreme variations in available nutrients, light, oxygen concentration, pressure, salinity, and temperature. Deep-sea organisms have developed unique biochemical metabolic and physiological capabilities, which not only ensure their survival in this habitat but also provide potential for the production of novel metabolites absent in terrestrial microorganisms (Fenical, 1993; Bull et al., 2000; Skropeta and Wei, 2014). Through molecular ecology studies, actinobacterial operational taxonomic units (OTUs) have been identified from deep sea sediments. Most of those foreseeably exhibit novel species, genera and families (Stach and Bull, 2005; Chen et al., 2016). Diverse species of actinomycetes cultured from the deep seafloor surface, including the deepest sea sediment samples from the Mariana Trench, have shown great biosynthetic capacities and thus a potent source of novel natural products (Pathom-aree et al., 2006d; Abdel-Mageed et al., 2010). With the breakthrough of technological barriers associated with deep sea actinobacteria isolation strategies, such as sample collection and cultivation under standard laboratory conditions, more and more deep sea actinobacteria and their natural products have been identified. Here we review the recent progress on deep sea actinomycetes and their metabolites from literature during year 2006–2016.

## Deep sea environment and biodiversity

The vast oceans cover 70% of the world's surface, with 95% greater than 1,000 m deep. Deep sea environments are divided into the bathyal zone (depths between 200 and 2,000 m), the abyssal (depths between 2,000 and 6,000 m) and the hadal zone (depths below 6,000 m) (Harino et al., 2009). Below sea level pressure is increased by depth, thereby in the deepest part of the trenches, the pressure varying from 10 atm at the shelf- slope interface to >1,000 atm. At bathyal depths temperatures taper off rapidly with increasing depth to 2°C. Deep-sea species must adjust their biochemical processes to survive in low temperatures, because the cold reduces chemical reaction rates. Oxygen concentration drops along with the depth, oxygen-minimum layer in mid-water, usually between 300 and 1,000 m depth. Light intensity decreases exponentially with depth in the water column. No photosynthetically useful light reaches the sea floor below about 250 m (Thistle, 2003).

Start at about 200 m depth, the deep sea is characterized by high pressure, low temperature, lack of light and variable salinity and oxygen concentration (Das et al., 2006), at the shelf break, where a clear change of fauna from shallow to deep water is observed (Thistle, 2003). According to Haefner (2003), in cold deep sea mud the diversity of life can be remarkably high with species richness rivaling that of tropical rain forest. Studying the species level of microbial diversity, finding a large number of rare species which more than half of them considered as new species and more than 95% is unidentified, furthermore the expanding of biodiversity reach to the 5,000 m in depth to abyssal which the peak amount of species at the depths of 3,000 m and beyond (Skropeta, 2008). On earth abyssal hills are the most abundant of biomass, but on wider abyss the ecological impact of the habitat heterogeneity is largely unexplored (Durden et al., 2015).

## Deep sea actinomycetes cultivation

However, so far only a few actinomycetes have been isolated from deep sea. It is because of technological barriers associated with isolation strategies. Therefore, we are in the pace to develop efficient cultivation methods to recover the actinobacteria population from extreme deep sea habitats. To achieve the task, firstly collection of samples from deep sea plays a pivotal role. In recent years several advancements have been developed in the context of sample collection from deep sea such as modified sediment grab and designer-built bounce corer (Fenical and Jensen, 2006), remote-operated submarine vehicle (Pathom-aree et al., 2006d), neuston sampling devices (Hakvåg et al., 2008), multi-core sampler (Xu et al., 2009), gravity or piston cores (D'Hondt et al., 2009), and untethered coring device (Prieto-Davó et al., 2013).

It is crucial to cultivate deep sea actinomyetes under standard laboratory conditions. There are several factors that influence the isolation, such as pre-treatment of dry heat (Shin et al., 2008), media composition (Luo et al., 2011; Pan et al., 2013; Song et al., 2015), dilution factor (Pathom-aree et al., 2006a), seawater requirement (Song et al., 2015), artificial seawater (Pan et al., 2013; Pesic et al., 2013) and incubation time (Song et al., 2015). It has also shown the addition of different antibiotics on selective media can inhibit the growth of fungal and bacterial contamination in order to enhance the actinomycetes growth similar to those used in isolation of actinomycetes from terrestrial sample. Long term freeze storage of deep sea sediment samples at −80°C has shown to prevent the growth of fast-growing bacteria which in results enhance the actinomycetes population (Ulanova and Goo, 2015). For the initial isolation of *Streptomyces*, cultivation temperatures have also influenced the recovery from deep sea sediment samples. Optimal growth temperature generally ranging from 25 to 30°C for successful cultivation of deep sea actinomycetes (Jeong et al., 2006; Luo et al., 2011; Pesic et al., 2013).

Heat pre-treatment procedures have been used effectively for the selective isolation of members of several actinomycete taxa and also inhibited growth of bacterial and fungal colonies. Moreover, actinomycete spores and hyphae are more sensitive to wet than dry heat hence relatively low temperature regimes are used to pretreat water and soil suspensions. Although heat pretreatment procedures decrease the ratio of bacteria to actinomycetes on isolation plates, the numbers of actinomycetes may also be reduced (Williams et al., 1972; Pathom-aree et al., 2006a,b,c,d). Pathom-aree et al., isolated actinomycetes from Norwegian fjord sediments support that the numbers of actinomycetes were reduced when used heat pretreatment for isolation; fewer actinomycetes were isolated on selective media inoculated with suspensions treated at 55°C as opposed to 50°C. Similarly, higher counts were generally recorded on isolation plates seeded with non-heat pretreated suspensions (Pathom-aree et al., 2006d).

For the other method, Jensen et al., 2005 used dry and stamp method for isolation actinomycetes from tropical Pacific Ocean and found that using this method for isolation of actinomycetes showed good recovery of 44%. In addition, Ulanova and Goo (2015) found that the majority of actinomycete-like colonies were also isolated using dry stamping technique from subseafloor sediments at the Nankai and Okinawa Troughs.

## Novel actinomycete species

Novel actinomycete species isolated from deep sea environment between 2006 and 2016, have yielded an impressive array of novel species with the highest number found at depths of abyssal zone and deeper. Different media has been used by researchers (Table 1). It is worth to be noticed that long time culturing and low temperature were employed for some of the novel isolates (Table 1). Only one novel *Microbacterium marinum* was obtained by pretreatment at 55°C, 6 min, others were from none heat pretreated samples (Table 1). The novel deep sea actinomycete species including 21 species under 13 genera with the maximum number from *Microbacterium* (*n* = 4), followed by *Dermacoccus* (*n* = 3), *Streptomyces* (*n* = 3) and *Verrucosispora* (*n* = 2), and one novel species for each of the other 9 genera (Table 1).

**Table 1:** **New actinomycetes species (***n*** = 21) isolated from deep sea environment between 2006 and 2015**.

**Species**	**Region**	**Depth(m)**	**Culture technique**			**References**
			**Extraction of act obact ria propagules/pretreatment procedure**	**Media**	**Incubation temperature and time**	
*Amycolatopsis marina* sp. nov.	South China Sea	Not sp cified	Not specified	SM1 with cycloheximide, neomycin sulfate and nystatin	28°C for 4 weeks	Bian et al., 2009
*Brevibacterium oceani* sp. nov.	Chagos Trench, Indian Ocean	5,904	Vortex sediment suspension in 2% NaCl for 1 min	Yeast extract/peptone (YP) agar	15°C for 15 days	Bhadra et al., 2008
*Dermacoccus abyssi* sp. nov.	Mariana Trench (Challenger Deep)	10, 898	Shaking sediment suspension for 30 min at 150 rpm	Raffinose-histidine agar with cycloheximide and nystatin	28°C for 12 weeks	Pathom-aree et al., 2006a
*Dermacoccus barathri* sp. nov.	Mariana Trench (Challenger Deep)	10, 898	Shaking sediment suspension for 30 min at 150 rpm	Raffinose-histidine agar with cycloheximide and nystatin	28°C for 12 weeks	Pathom-aree et al., 2006b
*Dermacoccus profundi* sp. nov.	Mariana Trench (Challenger Deep)	10, 898	Shaking sediment suspension for 30 min at 150 rpm	Raffinose-histidine agar with cycloheximide and nystatin	28°C for 12 weeks	Pathom-aree et al., 2006b
*Microbacterium indicum* sp. nov.	Chagos Trench, Indian Ocean	5,904	Vortex sediment suspension in 2% NaCl for 1 min	Yeast extract/peptone (YP) agar	15°C for 15 days	Shivaji et al., 2007
*Microbacterium marinum* sp. nov.	South-west Indian Ocean	2,800	Heated sediment suspension in a water bath at 55°C for 6 min	Modified DNB- seawater medium with nalidixic acid and nystatin	28°C for 1 week	Zhang L. et al., 2012
*Microbacterium profundi* sp. nov.	East Pacific polymetallic nodule region	5,280	Vortex sediment suspension in sterile seawater for 15 min	Modified ZoBell medium	25°C for 2 weeks	Wu et al., 2008
*Microbacterium sediminis* sp. nov.	South-west Indian Ocean	2,327	Vortex sediment suspension in sterile seawater	FJ sea water (50%) agar with rifampicin and potassiumdichromate	28°C	Yu et al., 2013
*Modestobacter marinus* sp. nov.	Atlantic Ocean	2,983	Not specified	Not specified	Not specified	Xiao et al., 2011b
*Myceligenerans cantabricum* sp. nov.	Avile's Canyon in the Ca tabrian Sea, Asturias, Spain	1,500	Not specified	1/3 tryptic soy agar and 1/6 M-BLEB sea water agar with cycloheximide and nystatin	28°C for 2 weeks	Vizcaíno et al., 2015
*Nesterenkonia alkaliphila* sp. nov.	Western Pacific Ocean	7,118	Not specified	Modified ISP 1- seawater	28°C for 3 weeks	Zhang et al., 2015
*Pseudonocardia antitumoralis* sp. nov.	South China Sea	3,258	Not specified	ISP 5- seawater medium	28°C for 3 weeks	Tian et al., 2013
*Sciscionella marina* gen. nov., sp. nov.	Northern South China Sea	516	Not specified	Gauze No. 1 -seawater medium	28°C for 3 weeks	Tian et al., 2009
*Serinicoccus profundi* sp. nov.	Indian Ocean	5,368	Not specified	Oligotrophic- seawater medium	Not specified	Xiao et al., 2011a
*Streptomyces indicus* sp. nov.	Indian Ocean	2,434	Not specified	Modified HV—sea water (75%) medium	25°C	Luo et al., 2011
*Streptomyces nanhaiensis* sp. nov.	South China Sea	1,632	Not specified	Humic acid-vitamin- sea water (70%) medium	28°C for 3 weeks	Tian et al., 2012a
*Streptomyces oceani* sp. nov.	Northern South China Sea	578	Not specified	10 % Nutrient seawater agar	28°C for 3 weeks	Tian et al., 2012b
*Verrucosispora maris* sp. nov.	Sea of Japan	Not specified	Not specified	Colloidal chitin agar	30°C for 4 weeks	Goodfellow et al., 2012
*Verrucosispora sediminis* sp. nov.	South China Sea	3,602	Not specified	Gauze No. 1 medium	22°C for 4 weeks	Dai et al., 2010
*Williamsia marianensis* sp. nov.	Mariana Trench (Challenger Deep)	10, 898	Shaking sediment suspension for 30 min at 150 rpm	Raffinose-histidine agar with cycloheximide and nystatin	28°C for 12 weeks	Pathom-aree et al., 2006c

SM1^*^: yeast nitrogen base (67.0 g; Difco) and casamino acids (100 mg; Difco) are added to a liter of distilled water and the solution sterilized using cellulose filters (0.20 mm) prior to the addition of sterilized dipotassium hydrogen phosphate (200 ml; 10%,w/v);100 ml of this basal medium was added to 900 ml of sterilized molten agar (1.5%,w/v) followed by filter ster l sed solutions of D (−) sorbitol (final concentration 1%,w/v); YP agar^*^ per liter distilled water: 5 g, yeast extract, 10 g peptone, 30 g NaCl, 15 g agar; Raffinose-h st d ne agar^*^: Raffinose 10 g, L-histidine 1 g, MgSO_4_ 7H_2_O 0.5 g, FeSO_4_ 7H_2_O 0.01g, K_2_HPO_4_ 1 g, Agar 20 g, pH 7.0-7.4; Modified DNB medium^*^: 0.1 g p ptone, 0.05 g b f xtract, 0.05 g NaCl, 1000 mL artificial seawater, pH 7.5; Modified ZoBell agar^*^: 19.45 g NaCl, 8.8 g MgCl_2_, 3.24 g Na_2_SO_4_, 1.8 g CaCl _2_, 0.55 g KCl, 0.16 g NaHCO_3_, 0.1 g f rric citrate pentahydrate, 80 mg KBr, 34 mg CsCl_2_, 22 mg H_3_BO_3_, 4.0 mg Na_2_SiO_3_, 2.4 mg NaF, 1.6 mg, NH_4_NO_3_, 8.0 mg Na _3_PO_4_, 0.5 g p pton, 0.1 g yeast extract, 20 g agar (pH 5.5, adjusted with HCl); FJ agar^*^: 1% glucose, 1% yeast extract, 1.5% agar, 50% seawater; 1/3 tryptic soy agar and 1/6 M-BLEB^*^: 1/3 tryptic soy agar (TSA, Merck) and 1/6 M-BLEB [9 g MOPS BLEB base (Oxoid) in 1 l Cantabrian Sea water], agar; Modified SP 1^*^: (1 L atural seawate, pH 10 final): 10 g glucose, 5 g peptone, 5 g yeast extract, 0.2 g MgSO_4_. 7H_2_O, 10 g NaHCO_3_, 27 g Na_2_ CO_3_ 10H_2_O and 15 g agar; ISP 5 medium^*^: L-asparagine (anhydrous basis) 1.0 g, Glycerol 10.0 g, K_2_HP0_4_ (anhydrous basis) 1.0 g, natural seawater 1. 0 l, Trace salts solution 1.0 ml Agar 20.0 g; SM3^*^: glucose 10 g, peptone 5 g, tryptone 3 g, NaCl 5 g, agar 15 g, distilled water, 1 l, pH 7.0; Gauze No. 1 medium^*^: Soluble starch 20.0 g, KNO_3_ 1.0 g, NaCl 0.5 g, MgSO_4_ x 7 H_2_O 0.5 g, K_2_HPO_4_ 0.5 g, FeSO_4_ x 7 H_2_O 10.0 mg, Agar 15.0 g, Sea water 1.0 L, Adjust pH 7.4; Oligotrophic- seawater medium^*^: Oligotrophic medium (seawater, 2.0% agar); Modified HV medium^*^: humic acid 1.0 g, KCl 1.7 g, FeSO_4_. 7H_2_O 0.01 g, Na_2_HPO_4_ 0.5 g, MgSO_4_. 7H_2_O 0.5 g, CaCO_3_ 0.02 g, thiamine 0.5 mg, nicotinic acid 0.5 mg, pantothenic acid 0.5 mg, p-aminobenzoic acid 0.5 mg, riboflavin 0.5 mg, vitamin B6 0.5 mg, inositol 0.5 mg, biotin 0.25 mg, water 250 ml, seawater 750 ml, agar 18 g, pH 7.2; Humic acid-vitamin agar^*^: Humic acid 2g, Asparagine 1 g, K_2_HPO_4_ 0.5 g, FeSO_4_ 7H_2_O 0.5 g, Agar 20 g, Sea-water 1000 ml, pH 7.0–7.4; 10% Nutrient agar^*^: Beef extract 0.03 g, peptone 0.05 g, agar 15 g, sea water 1 L; colloidal chitin agar^*^: 4 g of chitin, K_2_HPO_4_ (0.7 g); KH_2_PO, (0.3 g); MgSO_4_-5H_2_O (0.5 g); FeSO_4_.7H20 (0.01 g); ZnSO_4_ (0.001 g); MnCl_1_, (0.001 g); and 20 g of agar. pH 8.0

## Natural products synthesized by deep sea actinomycetes

The numbers of novel microbial metabolites from deep sea sediment samples have been increasing, especially from deep sea streptomycetes. Eight genera of actinomycetes were reported to produce secondary metabolites, among which *Streptomyces* is the richest producer (Table 2). Earlier culture dependent studies strongly suggested that *Streptomyces* species are present in considerable number in deep sea sediment samples (Jensen et al., 2005; Pathom-aree et al., 2006d). In addition several novel species of deep sea derived *Streptomyces* strains with distinct metabolites have been reported which indicates deep sea *Streptomyces* are really worth in the context of novel natural products discovery (Pan et al., 2015; Song et al., 2015).

**Table 2:** **Natural products synthesized by deep sea actinomycetes**.

**Strain**	**Compounds**	**Region**	**Depth (m)**	**Bioactivity**	**References**
*Dermacoccus abyssi*	Dermacozines A–G	Mariana Trench (Challenger Deep)	10, 898	Moderate cytotoxic activity against the leukemia cell line K562	Abdel-Mageed et al., 2010
*Dermacoccus abyssi*	Dermacozines H-J	Mariana Trench (Challenger Deep)	10, 898	Radical scavenging activity	Wagner et al., 2014
*Marinactinospora thermotolerans*	Marinacarbolines A–D, Indolactam alkaloids	South China Sea	3,865	Strong antiplasmodial activity	Huang et al., 2011
*Microbacterium sediminis* sp.nov.	Microbacterins A and B	South-west Indian Ocean	2,327	Significatnt inhibitory effects against a panel of human tumor cell	Liu D. et al., 2015
*Micromonospora* sp.	Levantilides A and B	Mediterranean	4,400	Anticancer	Gärtner et al., 2011
*Nocardiopsis alba* SCSIO 03039	Methoxyneihumicin	Indian Ocean	Not specified	Anticancer	Zhang et al., 2013
*Nocardiopsis* sp.	Nocardiopsins A and B	Coast of Brisbane, Australia	55	No activity	Raju et al., 2010
*Pseudonocardia* sp.	Pseudonocardians A–C	South China Sea	3,258	Anticancer, antibacterial activity	Li et al., 2011
*Serinicoccus profundi* sp. nov.	Indole alkaloid	Indian Ocean	5,368	Antibacterial activity	Yang et al., 2013b
*Streptomyces cavourensis* NA4	Bafilomycins B1 and C1	South China Sea	1,464	Antifungal Substances	Pan et al., 2015
*Streptomyces drozdowiczii* SCSIO 10141	Marformycins	South China Sea	1,396	Anti- infective	Zhou et al., 2014
*Streptomyces fungicidicus*	Diketopiperazines	Western Pacific	5,000	Antifouling products	Li et al., 2006
*Streptomyces lusitanus*	Grincamycins B–F	South China Sea	3,370	Anticancer	Huang et al., 2012
*Streptomyces niveus* SCSIO 3406	Marfuraquinocins	South China Sea	3,536	Cytotoxic, antibacterial activity	Song et al., 2013
*Streptomyces olivaceus* FXJ8.012	Tetroazolemycins A and B	Southwest Indian Ocean	Not specified	Metal ion-binding activity	Liu et al., 2013
*Streptomyces scopuliridis* SCSIO ZJ46	D sotamides B−D	South China Sea	3,536	Antibacterial activity	Song et al., 2014
*Streptomyces* sp.	Ammosamides A and B	Bahamas	1,618	Anticancer	Gaudêncio et al., 2008
*Streptomyces* sp.	Benzoxacystol	Atlantic	3,814	Inhibitory activity against the enzyme glycogen synthase kinase-3b	Nachtigall et al., 2011
*Streptomyces* sp.	Caboxamycin	Atlantic	3,814	Inhibitory activity against Gram-positive bacteria, anticancer	Hohmann et al., 2009
*Streptomyces* sp.	Spiroindimicins A–D	Indian Ocean	3,412	Anticancer	Zhang W. J. et al., 2012
*Streptomyces* sp.	Streptokordin	Ayu Trough	Not specified	Anticancer	Jeong et al., 2006
*Streptomyces* sp.	Streptopyrrolidine	Ayu Trough	Not specified	Anti-angiogenesis activity	Shin et al., 2008
*Streptomyces* sp. ACT232	Ahpatinin	Sagami Bay	1, 174	Aspartic protease inhibitors	Sun et al., 2014
*Streptomyces* sp. SCSIO 01127	Lobophorins E and F	South China Sea	1, 350	Antibacterial activity, cytotoxicity	Niu et al., 2011
*Streptomyces* sp. SCSIO 03032	Heronamides D−F	Indian Ocean	3,412	No activity	Zhang W. et al., 2014
*Streptomyces* sp. SCSIO 03032	Indimicins	Indian Ocean	3,412	Cytotoxic	Zhang W. J. et al., 2014
*Streptomyces* sp. SCSIO 04496	(6R,3Z)-3-benzylidene-6-isobutyl-1-methyl piperazine-2,5-dione	South China Sea	3,536	No activity	Luo et al., 2015
*Streptomyces* sp. SCSIO 10355	Strepsesquitriol	Indian Ocean	3,412	Inhibitory activity against lipopolysaccharide-induced TNFα production	Yang et al., 2013a
*Streptomyces* sp. SCSIO 11594	Dehydroxyaquayamycin	South China Sea	2,403	Antibacterial activity	Song et al., 2015
*Streptomyces* sp. SCSIO 11594	Marangucycline B	South China Sea	2,403	Anticancer	Song et al., 2015
*Streptomyces* sp. SNJ013	Sungsanpin	Jeju Island	138	Inhibitory activity to A549 with cell invasion assay	Um et al., 2013
*Streptomyces* sp. UST040711-290	12-methyltetradecanoid acid (12-MTA)	Pacific	5,774	Antifouling	Xu et al., 2009
*Streptomyces* sp. TP-A0873	Butenolids	Toyama Bay	Not specified	Peroxisome proliferator activated receptor—PPARα agonistic	Igarashi et al., 2015; Komaki et al., 2015
*Streptomyces* sp. 12A35	Lobophorins H and I	South China Sea	2,134	Antibacterial activity	Pan et al., 2013
*Streptomyces* strain C42	Champacyclin	Baltic Sea	241	Antimicrobial activity	Pesic et al., 2013
*Streptomyces xiamenensis* M1-94P	Xiamenmycin C and D	Pacific Ocean	2,628	Anti-fibrotic	You et al., 2013
*Verrucosispora* sp.	Abyssomicins J–L	South China Sea	2,733	Antibacterial activity	Wang et al., 2013

The deepest sea sediment samples from the Mariana Trench have been shown to possess great biosynthetic capacities. Seven dermacozines A–G were reported from the actinobacteria *Dermacoccus abyssi* sp. nov., strains MT1.1 and MT1.2 isolated from Mariana Trench sediment collected at a depth of 10 898 m. Dermacozines F and G displayed moderate cytotoxic activity against the leukemia cell line K562 with IC_50_ values of 9 and 7 mM, respectively, whereas dermacozine C also exhibited high radical scavenger activity with an IC_50_ value of 8.4 mM (Abdel-Mageed et al., 2010).

In recent years, South China Sea has been emerging as a potentially abundant source of novel species/genera of marine actinomycetes. Some bioactive compounds, such as pseudonocardians A-C, grincamycins B-F, and abyssomicins J-L were reported. Natural products derived from deep sea actinomycetes discovery have displayed a wide range of bioactivities, such as antitumor, antimicrobial, antifouling, and anti-fibrotic activities (Table 2).

## Biosynthesis pathways for deep sea streptomycetes natural products

Lobophorins H and I together with three known analogs, *O*-β-kijanosyl-(1 → 17)-kijanolide, lobophorins B and F were yielded by *Streptomyces* sp. 12A35, isolated from a deep sea sediment sample collected at a depth of 2,134 m in South China Sea (Pan et al., 2013). While, lobophorins E and F, along with two known analogs lobophorins A and B were discovered from the products of the deep sea *Streptomyces* sp. SCSIO 01127, was isolated from sample collected at a depth of 1,350 m in the South China Sea (Niu et al., 2011). The gene cluster involved in biosynthesis of lobophorin was the first type I PKS gene cluster identified from the deep sea derived *Streptomyces*. Three glycosyltransferases (GTs) LobG1-LobG3 genes-inactivation mutants yielded five different glycosylated metabolites, and the result suggested that LobG3 as an iterative GT to attach two L-digitoxoses (Li et al., 2013). Desotamides B, C and D together with a known desotamide A were obtained from deep sea derived *Streptomyces scopuliridis* SCSIO ZJ46, recovered from sediment sample collected at a depth of 3,536 m in the South China Sea (Song et al., 2014). A 39 kb gene cluster governing the biosynthesis of the anti-infective desotamides has been isolated from the strain. Desotamides A and B and a new desotamide G have been obtained by heterologous expression of desotamide gene cluster in *Streptomyces coelicolor* M1152 (Li et al., 2015).

Heronamides D, E, and Fare discovered from the products of *Streptomyces* sp. SCSIO 03032, which was isolated from deep sea sediment sample collected at a depth of 3,412 m in the Bay of Bengal, Indian Ocean (Zhang W. et al., 2014). The gene cluster governing the biosynthesis of heronamide has been isolated from strain SCSIO 03032. The gene inactivation study confirmed that P450 enzyme encode HerO as an 8-hydroxylase for tailoring heronamide biosynthesis. Feeding experiments with labeled small carboxylic acid molecules confirmed the migrated double bonds in the conjugated diene-containing side chain of heronamides (Zhu et al., 2015).

Marformycins A-F were obtained from fermentation broth of deep sea sediment-derived *Streptomyces drozdowiczii* SCSIO 1014, which was isolated from sample collected at a depth of 1,396 m in South China Sea. All compounds exerted selective anti-microbial activity against *Micrococcus luteus, Propionibacterium acnes*, and *P. granulosum*. Marformycins A-E displayed inhibitory activity against *M. luteus* with MICs of 0.25, 4.0, 0.25, 0.063, and 4.0 μg/mL, respectively, while they did not displayed any cytotoxicity (Liu D. et al., 2015). It is suggested that these compounds may be used as promising candidatures for anti-infective drug leads. The gene cluster that responsible for the biosynthesis of marformycin is about 45 kb in size and has been identified from strain SCSIO 10141. The gene inactivation studies indicated that three NRPS proteins MfnC, MfnD, MfnE, a free adenylation (A) enzyme MfnK, and a free peptidyl carrier protein (PCP) MfnL were essential for the generation of the marformycin core scaffold. Further, MfnN was found to use an intact cyclodepsipeptide intermediate as its substrate (Liu J. et al., 2015).

## Perspective

The discovery of novel actinomycete taxa with unique metabolic activity from deep sea samples, and novel compounds with the greatest biogenic, metabolic diversity and biological activities clearly illustrate that indigenous deep sea actinomycetes indeed exist in the oceans and are an important source of novel secondary metabolites. Other function of deep sea actinobacteria is also interesting such as oil degradation and biosurfactant production (Wang et al., 2014). It is worth to be noticed that no heat pretreatment, dry and stamp method and low temperature incubation were more productive for actinomycetes isolation from some deep sea samples. With the development of culture independent techniques, more productive strategy of strain isolation guided by the deep sea actinomycetes distribution or direct cloning and heterologous express the functional genes could be approached.

## Author contributions

MK contribute the introduction, deep sea environment and biodiversity, actinomycete cultivation, novel taxa, and Table 1. PS contribute sample collection, Table 2 and biosynthesis of secondary metabolites from deep sea streptomycetes. KH and ZD conceived the idea and revised the whole manuscript.

### Conflict of interest statement

The authors declare that the research was conducted in the absence of any commercial or financial relationships that could be construed as a potential conflict of interest.
